# Psychological Indices and Health‐Related Quality of Life in Renal Replacement Therapy: A Three Groups Cross‐Sectional Study

**DOI:** 10.1155/ijne/5976864

**Published:** 2025-12-22

**Authors:** Maryam Emadzadeh, Negar Morovatdar, Mohammad Javad Mojahedi, Majid Reza Erfanian Taghvaei, Fatemeh Moharreri, Fatemeh Nazemian, Ali Emadzadeh, Mohammad Khajedaluee

**Affiliations:** ^1^ Clinical Research Development Unit, Ghaem Hospital, Mashhad University of Medical Sciences, Mashhad, Iran; ^2^ Metabolic Syndrome Research Center, Mashhad University of Medical Sciences, Mashhad, Iran; ^3^ Clinical Research Development Unit, Imam Reza Hospital, Mashhad University of Medical Sciences, Mashhad, Iran; ^4^ Kidney Transplantation Research Center, Mashhad University of Medical Sciences, Mashhad, Iran; ^5^ Department of Community Medicine, Mashhad University of Medical Science, Mashhad, Iran, mums.ac.ir; ^6^ Department of Psychiatry, Psychiatry and Behavioral Sciences Research Center, Mashhad University of Medical Science, Mashhad, Iran; ^7^ Department of Nephrology, Department of Internal Medicine, Imam-Reza Hospital, Mashhad University of Medical Sciences, Mashhad, Iran; ^8^ Department of Internal Medicine, Faculty of Medicine, Mashhad Medical Sciences, Islamic Azad University, Mashhad, Iran, azad.ac.ir

**Keywords:** anxiety, depression, dialysis, quality of life, renal insufficiency

## Abstract

**Background:**

Chronic kidney disease (CKD) is a significant global health concern. Patients in the last stage of CKD, also known as end stage kidney disease (ESKD), need to use one of the methods of renal replacement therapy including hemodialysis (HD), peritoneal dialysis (PD), and kidney transplantation (KT). ESKD adversely affects physical and mental health, as well as overall quality of life. However, limited research has explored the association between these health burdens and treatment modalities in Iran.

**Methods and Materials:**

This cross‐sectional study included 215 patients with ESKD undergoing HD (*n* = 66), PD (*n* = 70), and KT (*n* = 79) at treatment centers in Mashhad, Iran. A checklist of demographic information, quality of life questionnaire specific for kidney disease patients (Kidney Disease Quality of Life Short Form [KDQOL‐SF], version 1.3), Hamilton depression and anxiety inventories, and the Cassidy inventory for problem‐solving and social support were distributed among patients. A regression model was employed to adjust for potential confounders, and statistical analysis was conducted using SPSS 11.5.

**Results:**

Kidney transplant recipients were younger than patients in the HD and PD groups and exhibited significantly higher mean scores in most domains of the KDQOL, both in general and specific domains. After adjusting for confounding variables such as age, transplant patients were found to have the highest quality of life scores in specific domains. Depression prevalence was high, ranging from 60% to 65% across all groups, with no significant intergroup differences. Anxiety prevalence ranged from 21% to 26%, also without significant differences. Social support was reported as high among ESKD patients (70%–82%) with no variation between treatment modalities.

**Conclusion:**

KT was associated with better quality‐of‐life outcomes compared to HD and PD, highlighting the potential benefits of organ donation programs. Given the substantial burden of mental health issues among ESKD patients, early detection and intervention for depression and anxiety should be prioritized in both dialysis and transplant populations.

## 1. Introduction

Chronic kidney disease (CKD) is a significant global public health issue, characterized by the progressive decline of kidney function, primarily due to hypertension and diabetes [1]. Disability‐adjusted life years (DALYs) for CKD have risen globally, moving from 29th place in 1990 to 18th place in 2019 [2]. A recent study in Yazd, Iran, revealed that the prevalence of CKD in the general population exceeded 27% [3]. CKD is considered one of the main causes of premature mortality, as well as mental and psychological side effects. It contributes significantly to years of life lost from mortality (YLL) and DALYs [4].

End stage kidney disease (ESKD) is the last stage of CKD. Renal replacement therapies (RRT), including hemodialysis (HD), peritoneal dialysis (PD), and kidney transplantation (KT), are the mainstay treatment options, help in the ESKD patients’ improvement and slow down disease progression. ESKD and its associated treatments profoundly affect both physical and mental well‐being, influencing overall quality of life (QoL). According to the International Society of Nephrology, 2.62 million individuals with ESKD received RRT worldwide, with the majority undergoing HD [5]. The main complications of HD include hypotension, muscle cramps, and anaphylactic reactions. In addition to these physical complications, HD patients also experience significant psychological distress and financial burdens related to treatment [6]. Similarly, PD patients face considerable social and emotional difficulties, with some studies reporting lower mental health‐related quality‐of‐life scores among this group [7]. A comparison of RRT modalities in Iran and globally indicates that the KT rate in Iran exceeds the international average. Worldwide, approximately 20% of ESKD patients undergo KT, whereas in Iran, this proportion rises to 50% [8–10]. Accessibility of KT varies globally ranging from 23% to 89% across low‐ and high‐income countries, and even within those categories. The availability of KT for more than half of the eligible patient population fluctuates from less than 1%–57% depending on economic conditions [11].

As CKD progresses, patients experience an increase in somatic symptoms, which contribute to reduced health‐related QoL and limitations in daily activities. In 2023, Kalantari et al. found that PD patients reported a better QoL compared to HD patients. The authors concluded that physicians should encourage the use of PD where feasible [12]. Although QoL has received increased attention in recent years, there remains limited research on the QoL of ESKD patients in developing countries. It is important to recognize that QoL assessment in ESKD patients is of great importance in developed countries and is evaluated as a main outcome in so many studies, leading to a new approach for these patients [13]. In addition to physical comorbidities such as cardiovascular diseases, musculoskeletal disorders, and lung conditions [14], CKD patients also experience psychological distress. In a recent meta‐analysis, the prevalence of anxiety disorders and symptoms among patients with CKD was 19% and 43%, respectively [15]. CKD patients with psychological disorders exhibited a higher risk of hospitalization and mortality [16]. A study conducted in Iran found the prevalence of anxiety and depression symptoms in CKD patients to be 13% and 66%, respectively [17]. Given that CKD is a chronic condition, adherence to treatment is crucial for effective disease management and the prevention of adverse outcomes and comorbidities. Unfortunately, non‐adherence to medication in common among CKD patients [18]. Furthermore, poor social support has been associated with reduced medication adherence and worse clinical outcomes [19].

Since few studies have compared psychiatric symptoms, social support, and QoL using a specific questionnaire across the three RRT modalities‐HD, PD, and KT‐this study aims to address these gaps. This “three‐group study” is the first of its kind in the region and seeks to shed light on psychosocial aspects of ESKD patients that may have been overlooked in prior research.

## 2. Methods and Materials

This analytical cross‐sectional study was conducted on ESKD patients undergoing three methods of RRT (HD, PD, and transplantation) in Mashhad, Iran. The ethical committee of Mashhad University of Medical Sciences approved the study (code: IR.MUMS.fm.REC.1395.12, approve date: 23rd May 2015). This study complies with the Declaration of Helsinki and Iranian Guidelines for Medical and Biological Research Involving Human Subjects.

### 2.1. Participants

Patients aged 14 and older without a confirmed diagnosis of a mental illness were included in this study. Patients who were referred to Imam Reza and Ghaem hospitals, as well as the kidney foundation of Iran for HD and PD, and those who were referred to Montaseriyeh hospital for post‐transplantation visits were recruited for the study. All participants included in the study were selected from outpatients who had been referred to the aforementioned hospitals for dialysis treatments or postoperative visits. Questionnaires were completed in person by two trained nurses under the supervision of a physician. Participants were required to have been receiving specific treatment with RRT for at least 6 months; otherwise, they were excluded from the study. Informed consent was obtained from all participants, and they were assured of the confidentiality of their personal information. Furthermore, the study objectives and methods were described to pediatric participants and their parents or guardians. After obtaining approval for their participation in the study, parents/guardian signed the informed consent forms, and questionnaires were distributed for completion.

The sample size was determined based on the findings of a study conducted by Zeraati et al. [20]. They reported that the general health (GH) subscale of QoL in HD patients was 39.06 ± 16.86, and in PD patients it was 50.87 ± 25.26. Taking into account the type II error (β) of 0.2 and a type I error (α) of 0.05, the minimum sample size was calculated to be 52 in each study group.

### 2.2. Measures

Sociodemographic and clinical checklists were distributed among all participants. Trained interviewers asked questions and completed the questionnaire for illiterate patients.

To evaluate the QoL in ESKD patients, we utilized the Kidney Disease QoL Short Form 1.3 (KDQOL‐SF). The reliability and validity of the Persian version of this questionnaire had been previously confirmed [21, 22]. The recent short form of this questionnaire (KDQOL‐SF^1^, version 1.3) is divided into two parts. The first part is identical to the SF‐36 QoL questionnaire, containing 36 questions across eight domains (physical functioning [PF], role‐physical, bodily pain (BP), GH, vitality, role‐emotional (RE), social functioning, and fatigue/energy) and one question regarding current health status compared to the previous year.

The second part of the questionnaire includes 43 questions. This section is specific to kidney disease QoL and contains questions related to eight main categories: symptoms/problems, effects of kidney disease on daily life, burden of kidney disease, work status, cognitive function, quality of social interaction, sexual function, and sleep. Additionally, there are three extra questions about social support, dialysis staff encouragement, and patient satisfaction.

Hamilton’s Depression Inventory was used to assess depression symptoms. This inventory consists of 24 questions with scores ranging from 0–2 or 0–4. The total score was calculated by adding up all the scores, and then categorized as follows: ≤ 7 (normal), 8–13 (mild), 14–18 (moderate), 19–22 (severe), and ≥ 23 (very severe) [23, 24].

Hamilton Anxiety Inventory was used to assess anxiety levels in the participants. This inventory includes 13 questions divided into two domains: mental and physical anxiety symptoms. Each question is rated on a scale of 0–4. A total score of ≤ 14 indicates no anxiety, while scores of 15–24, 25–40, and ≥ 40 indicate mild, moderate, and severe anxiety, respectively [25, 26].

Social support was evaluated using the Cassidy inventory for problem‐solving and social support. Scores of 0–4, 5–9, and 10–14 were classified as weak, moderate, and good social support [27–30]. It is important to note that the validity and reliability of all questionnaires used in this study were previously confirmed in Persian, and valid Persian inventories were utilized for this project.

### 2.3. Statistical Analysis

After collecting data, the data were analyzed using SPSS (version 11.5). In all analyses, a *p* value of less than 0.05 was considered significant. We utilized appropriate frequency tables and central and dispersion parameters to describe the findings. The chi‐square test was employed for categorical variables. The normal distribution of data was assessed using the Kolmogorov–Smirnov test. For normally distributed data, we used the analysis of variance (ANOVA) test to compare continuous variables among the three study groups. If the data were not normally distributed, the Kruskal–Wallis test was used. We used a generalized linear model to adjust for confounding variables such as age, income, and body mass index (BMI) that may impact the response variables.

## 3. Results

A total of 215 patients participated in our study. The distribution of patients across the three therapeutic groups was as follows: 66 (30.7%) in the HD group, 70 (32.6%) in the PD group, and 79 (36.7%) in the transplantation group. Additional descriptive data are provided in Table 1. As shown in this table, significant differences were observed across the three groups in terms of mean age, marital status, BMI, income level, history of tobacco use, and daily cigarette consumption.

**Table 1:** Participants’ characteristics in each study group.

Characteristics		HD (*n* = 66)	PD (*n* = 70)	Transplantation (*n* = 79)	*p* value
Sex	Male	33 (50)	30 (45.5)	39 (50.6)	0.77^a^
	Female	33 (50)	36 (54.5)	38 (49.4)	
Age		50.5 ± 16.39 [48 (38.5, 63)]	50.46 ± 18.24 [52 (32.5, 65)]	38.17 ± 12.89 [37 (29, 46)]	< 0.001^a^
Marital status	Single	14 (21.5)	10 (15.2)	29 (36.7)	< 0.01^a^
	Married	46 (70.8)	46 (69.7)	48 (60.8)	
	Widow	3 (4.6)	8 (12.1)	0 (0)	
	Divorced	2 (3.1)	2 (3)	2 (2.5)	
Education	Illiterate	14 (21.2)	13 (18.8)	10 (12.7)	0.62^b^
	Elementary school	13 (19.7)	16 (23.2)	24 (30.4)	
	High school	9 (13.6)	9 (13)	20 (25.3)	
	Diploma	17 (25.8)	18 (26.1)	19 (24.1)	
	Associate diploma	6 (9.1)	7 (10.1)	2 (2.5)	
	BS and higher	7 (10.6)	6 (8.7)	4 (5.1)	
Income (10000 IRR^c^)	Individual	1057.89 ± 688.25 (1000 [600, 1500])	501.72 ± 520.73 (300 [0, 1000])	434.15 ± 453.24 (500 [0, 700])	< 0.01^b^
	Family	967.02 ± 651.28 (1000 [600, 1000])	1081.13 ± 507.18 (1000 [700, 1500])	645.90 ± 440.29 (600 [400, 900])	< 0.001^b^
BMI		22.72 ± 3.77 (22.6 (20.15, 24.64])	25.06 ± 3.14 (25.24 [23.05, 27, 52])	23.45 ± 4.53 (23.08 [19.8, 26, 46])	< 0.01^d^
PMH	Heart disease	14 (21.2)	7 (10)	10 (12.7)	0.15^a^
	Respiratory disease	3 (4.5)	0 (0)	2 (2.5)	0.23^a^
	DM	19 (28.8)	29 (41.4)	10 (12.7)	< 0.001^a^
	HTN	20	40	12	< 0.001^a^
Tobacco use	Positive history	5 (7.6)	21 (30)	5 (6.3)	< 0.001^a^
	Duration (month)	144 (187.06)	87.75 (49.8)	320 (288.33)	0.31^d^

*Notes:* Data are presented as both mean ± standard deviation and (median [interquartile range]) for quantitative variables and frequency (percent) for qualitative variables.

Abbreviations: BMI, body mass index; PMH, past medical history; DM, diabetes mellitus; HTN, hypertension.

^a^Chi‐square.

^b^Kruskal–Wallis.

^c^IRR is the currency code for the Iranian Rial, the currency of Iran. Note that during the time of the study, each USD was valued at approximately 33,250 IRR.

^d^ANOVA.

The age of participants ranged from 15 to 87 years. A total of 31 patients (16.6%) reported a positive family history of kidney disease. Diabetes and hypertension were identified as the leading causes of kidney disease in 13.6% and 25.8% of patients, respectively. While the transplantation group exhibited the lowest smoking history, patients in this group reported the longest duration of smoking and the highest daily cigarette consumption (Table 1). Among all participants, seven had a history of substance abuse, with four in the transplantation group and three in the PD group.

Table 2 presents data on various dimensions of QoL, encompassing both GH indicators and aspects specific to CKD. As shown in Table 2, panel A, significant differences were observed in mean percentage scores across five domains: PF, BP, GH, vitality, and fatigue/energy. Transplant recipients exhibited the highest scores in all areas except for the physical and emotional role domains. The lowest scores for PF, BP, RE, and fatigue/energy were observed in the PD group, whereas HD patients had the lowest scores in the remaining domains. After adjusting for age, income, and BMI within the generic scales of the SF‐36 domains, transplant patients consistently demonstrated superior QoL in the BP, GH, and vitality domains. Age was a significant predictor for all domains except for vitality and RE (Table S1).

**Table 2:** Quality of life in three therapeutic groups.^a^

**Panel A. Generic scales of SF-36 questionnaire**				
	**HD (*n* = 66)**	**PD (*n* = 70)**	**Transplantation (*n* = 79)**	***p* value**

Physical functioning (PF)	52.22 (30.42)	51.34 (25.97)	69.55 (23.75)	< 0.001^b^
Role‐physical (RP)	25.58 (41)	33.48 (39.1)	31.5 (37.73)	0.28^b^
Bodily pain (BP)	60.07 (28.07)	53.89 (27.31)	76.17 (24.87)	< 0.001^b^
General health (GH)	47.5 (16.62)	49.1 (19.67)	58.03 (17.1)	0.001^b^
Vitality	60.31 (18.5)	61 (18.61)	67.79 (21.35)	0.04^c^
Role‐emotional (RE)	53.33 (47.06)	39.52 (42.19)	47 (42.1)	0.13^b^
Social functioning (SF)	54.73 (26.56)	57.06 (22.63)	61.53 (27.08)	0.27^b^
Fatigue/energy	55.93 (20.46)	51.71 (18.68)	64.87 (22.53)	0.001^c^

**Panel B. Specific scales of KDQOL-SF**				
	**HD (*n* = 66)**	**PD (*n* = 70)**	**Transplantation (*n* = 79)**	***p* value**

Symptom/problems	68.08 (14.56)	65.75 (13.99)	73 (15.45)	< 0.01^b^
Effects of kidney disease on daily life	59.31 (19.42)	66.78 (20.45)	78.46 (15.99)	< 0.001^c^
Burden of kidney disease	39.01 (24.85)	30 (23.97)	45.64 (25.36)	< 0.001^b^
Work status	26.15 (25.16)	21.42 (28.95)	17.08 (28.74)	0.04^b^
Cognitive function	60.6 (14.21)	55.65 (17.99)	62.9 (20.37)	0.01^b^
Quality of social interaction	62.08 (16.87)	55.25 (22.48)	65.23 (21.68)	0.01^c^
Sexual function	58.84 (33.68)	50.33 (29.23)	77.27 (24.89)	0.001^b^
Sleep	67.13 (18.81)	58.87 (17.59)	70.97 (22.79)	0.001^c^
Social support	67.94 (24.17)	69.84 (23.44)	74.25 (28.21)	0.1^b^
Dialysis staff encouragement	76.2 (30.17)	87.67 (15.85)	82.78 (10.95)	0.02^b^
Patient satisfaction	72.97 (24.06)	57.48 (21.3)	69.23 (24.33)	< 0.001^b^

*Note:* Tukey’s test in SF‐36 indicated a difference between the transplantation and two other groups in subscales with significant *p* value, except in the *vitality* which Tukey’s test could not find the source of difference. Tukey’s test in KDQOL‐SF indicated a difference between the transplantation and PD groups in all subscales. Only in the subscales of *dialysis staff encouragement* and *patient satisfaction* the PD group was the source of difference.

Abbreviation: KDQOL‐SF, Kidney Disease Quality of Life; Short Form.

^a^For ease of score comparison, all scores were calculated as percentages. The data are presented as mean (SD).

^b^Kruskal–Wallis.

^c^ANOVA.

Table 2, panel B, illustrates the specific aspects of QoL in kidney disease using KDQOL‐SF 1.3. There are significant differences in all domains of QoL (except social support) among the three treatment groups. The PD group reported the highest mean score in the Dialysis Staff Encouragement domain, while the HD patients attained the highest scores in work status and patient satisfaction. In all remaining domains, transplant recipients demonstrated the highest mean scores. After adjusting for age, income, and BMI, significant differences among groups persisted in the effects of kidney disease on daily life and sexual function domains. We also found that age had a reverse association with symptoms, daily life, work status, and sleep QoL domains (Table S2).

A total of 101 (48.3%) patients reported that their health status was better than the previous year. The majority of these patients were transplant recipients (51 patients). On the other hand, eight participants (3.8%) believed that their health status was much worse than the previous year, with 1, 3, and 4 patients belonging to the transplantation, PD, and HD groups, respectively. The statistical test showed a significant relationship between patients’ responses and their therapeutic groups (*p* = 0.001) (Table 3).

**Table 3:** Patients’ health status compared to the previous year.

Health status	HD (*n* = 66)	PD (*n* = 70)	Transplantation (*n* = 79)	*p* value
Much better	9 (14.5)	8 (11.8)	34 (43)	< 0.001^a^
Somewhat better	14 (22.6)	19 (27.9)	17 (21.5)	
About the same	17 (27.4)	20 (29.4)	12 (15.2)	
Somewhat worse	18 (29)	18 (26.5)	15 (19)	
Much worse	4 (6.5)	3 (4.4)	1 (1.3)	

*Notes:* Data presented as frequency (percentage).

Abbreviations: HD, hemodialysis; PD, peritoneal dialysis.

^a^Kruskal–Wallis.

The frequency of different levels of depression in patients with HD, PD, and those who underwent transplantation was 65%, 64%, and 60%, respectively. We found that anxiety levels were lower among the study population compared to depression frequency, with rates of 21% in HD, 26% in PD, and 24% in transplanted patients. We did not find any statistical differences in depression and anxiety levels among the study groups (Table 4).

**Table 4:** Anxiety and depression status according to Hamilton scales among study groups.

Levels of depression	HD (*n* = 66)	PD (*n* = 70)	Transplantation (*n* = 79)	*p* value
Normal	23 (34.8)	25 (35.7)	32 (40.5)	0.96^a^
Mild mood disturbance	20 (30.3)	18 (25.7)	19 (24.1)	
Moderate depression	10 (15.2)	13 (18.6)	11 (13.9)	
Severe depression	10 (15.2)	7 (10)	1 (1.3)	
Extreme depression	3 (4.5)	7 (10)	16 (20.3)	
Levels of anxiety	HD (*n* = 66)	PD (*n* = 70)	Transplantation (*n* = 79)	*p*‐value
Normal	52 (78.8)	52 (74.3)	60 (76)	0.76^a^
Low anxiety	12 (18.2)	13 (18.6)	12 (15.2)	
Moderate anxiety	2 (3)	5 (7.1)	6 (7.6)	
Severe anxiety	0 ()	0 ()	1 (1.3)	

Data presented as frequency (percentage).

Abbreviations: HD, hemodialysis; PD, peritoneal dialysis.

^a^Kruskal–Wallis.

We found fairly high levels of social support among the study population (82% in HD, 70% in PD, and 81% in transplanted patients). Additionally, we found no difference in social support levels among the study groups (data not shown). Moreover, we assessed the correlation between social support and depression and anxiety. The results showed that social support has a weak correlation with depression (*r* = −0.25, *p* < 0.001). No significant correlation was found between social support and anxiety (*r* = −0.12, *p* = 0.08).

## 4. Discussion

This study demonstrated that transplant recipients achieved higher scores in multiple QoL domains, including BP, GH, vitality, effects of kidney disease on daily life, and sexual function, indicating superior overall QoL. Besides treatment modality, age emerged as a key factor influencing QoL, with older patients reporting lower scores. No significant differences were observed among the three groups regarding depression and anxiety symptoms. Notably, over 70% of patients in each group reported adequate social support.

Among the sociodemographic characteristics, transplant patients were approximately 10 years younger than those undergoing HD or PD, consistent with previous studies by Namadi and Abbaszadeh, which reported transplant recipients being 10 and 5 years younger than dialysis patients, respectively [31, 32].

Our study revealed significant differences in most SF‐36 QoL domains across the three treatment groups, with transplant patients consistently exhibiting the highest scores. Within this group, the highest QoL scores were observed in the pain domain, whereas the lowest were in physical problems. This aligns with Sitjar‐Suner et al., who identified *PF* as the only domain to decline over a 5‐year longitudinal study [33]. Most Iranian studies, including those by Namadi [32] and Taghizadeh [34], corroborate that transplant patients have better QoL than dialysis patients. However, Harirchi’s 2003 study was an exception, reporting lower QoL scores in transplant recipients compared to HD patients. This discrepancy could be attributed to the type of questionnaire that he utilized. He administered the *Evans and Cope* questionnaire, in which patients responded to the inquiries by selecting yes or no. Notably, this questionnaire also evaluated political, creative, and artistic tendencies. Additionally, Harirchi’s study included only male participants [35].

After adjusting for potential confounders, several QoL domains remained significantly better in transplant patients compared to HD patients. Although the younger mean age of transplant recipients could partly explain their better QoL [36–38], adjustment for age did not fully account for these differences, suggesting that transplantation itself confers QoL benefits. Similar findings have been reported in other studies, both before and after age adjustment [39, 40]. Notably, the adjusted analyses showed smaller differences between treatment modalities than unadjusted results, indicating that QoL in renal patients depends not only on treatment modality but also on other factors such as age.

A systematic review showed that kidney transplant patients tend to engage more in daily activities, physical exercise, jobs, and journeys. Interestingly, these results were consistent across all studies [41]. This supports our observation of superior QoL in transplant recipients. Ramus et al. found comparable QoL between HD and PD patients, with PD patients reporting less pain [42]. In the study conducted by So et al., 67 percent of HD participants experienced some degree of pain or discomfort in the related domain of the EQ5D5L (EuroQOL) questionnaire [43]. However, in our study, PD patients reported the worst pain in the QoL inventory. It should be noted that men were dominant in the So et al. study, while in our study, the frequency of women in the PD group was higher than that of men. This could justify the higher recorded pain in this group. Moreover, Aseel et al. indicated that pain has a significant negative effect on the QoL of ESKD patients. He also stated that females were at a higher risk [44].

Jaberi et al. identified a negative correlation between QoL and death anxiety among HD patients, although they only assessed death anxiety specifically [45]. A retrospective cohort study in Australia found that anxiety and depression symptoms decreased over 12 months across PD, HD, and transplant groups, with the greatest reduction observed in PD patients [33]. Moreover, at the beginning of the study, over half of the PD patients reported some degree of depression or anxiety [33]. In the current study, approximately 35% and 75% of participants in each study group did not exhibit any symptoms of depression or anxiety, respectively. As noted by Hao et al., these symptoms may be influenced by other clinical and sociodemographic variables [46].

Social support could predict the mental health status of human beings [47]. Some studies have examined social support in CKD patients and its relationship with psychological status [45, 48, 49]. In our study, all three groups showed good social support. This finding aligns with the results of Asadizaker et al., who also found a negative association between social support and depression [48]. However, Jaberi did not find a significant relationship between social support and death anxiety [45]. Gunarathne et al. highlighted that high stress status and poor social support contribute to symptom burden in CKD patients. It is important to highlight that all of these studies were conducted on HD patients [49].

The strengths of this study include assessing outcomes in a broader range of patients undergoing CKD treatment, whereas in most previous articles, QoL was only evaluated in one or two treatment groups. Additionally, we examined the QoL, social support, and mental health status of these patients. In earlier studies, these three parameters were often overlooked. The limitations of our study listed here; First, as this is a cross‐sectional study, it does not allow for conclusions about temporality or causality, and no follow‐up data are available to determine whether participants remained on RRT at the time of analysis. Second, all participants were recruited from outpatient settings—specifically individuals referred for dialysis treatments or postoperative follow‐up visits—which introduces a potential selection bias. Third, the study did not collect information on previous ICU admissions, a factor that could act as a relevant confounder in the interpretation of psychological outcomes. Additionally, variations in questionnaire administration may have influenced the results. Questionnaires for the HD group were completed during HD, while the KT patients completed their questionnaires during postoperative visits. Some studies on physical activity suggest that the level of routine activities predicts both QoL and disease outcomes. Therefore lastly, the lack of information regarding patients′ physical activity levels and their adherence to treatment in the current study represents a potential limitation [50, 51]. These differing circumstances could introduce bias; however, they were unavoidable.

## 5. Conclusion

According to the current study, transplant patients have a better QoL compared to other treatment options for RRT. Prevention strategies for slowing down the progression of kidney disease are crucial for improving the QoL of kidney patients, especially in low‐ and middle‐income countries where resources for transplantation are limited. Screening and managing the high prevalence of depression among CKD patients is essential. Interventional studies, including psychological and social support consultations, should be conducted not only in dialysis patients but also in transplant patients.

## Conflicts of Interest

The authors declare no conflicts of interest.

## Author Contributions

Conceptualization: Maryam Emadzadeh, Mohammad Khajedaluee, Ali Emadzadeh. Formal analysis: Mohammad Khajedaluee, Maryam Emadzadeh. Funding acquisition: Mohammad Khajedaluee. Investigation: Maryam Emadzadeh. Project administration: Mohammad Khajedaluee, Mohammad Javad Mojahedi, Majid Reza Erfanian Taghvaei, Fatemeh Moharreri, Fatemeh Nazemian. Writing–original draft: Maryam Emadzadeh, Negar Morovatdar. Writing–review and editing: Maryam Emadzadeh, Negar Morovatdar, Mohammad Khajedaluee, Ali Emadzadeh. Maryam Emadzadeh and Negar Morovatdar are contributed equally as the first author.

## Funding

This study was supported by the Mashhad University of Medical Sciences (no. 930254).

## Endnotes

^1^Kidney Disease Quality of Life Short Form (KDQOL‐SF), version 1.3.

## Supporting Information

Table S1: Adjusted regression models for generic scales of SF‐36 questionnaire.

Table S2: Adjusted regression models for specific scales of KDQOL‐SF.

## Supporting information


**Supporting Information** Additional supporting information can be found online in the Supporting Information section.

## Data Availability

The data that support the findings of this study are available on request from the corresponding author. The data are not publicly available due to privacy or ethical restrictions.
