# Supplementary material for: Psychological Indices and Health‐Related Quality of Life in Renal Replacement Therapy: A Three Groups Cross‐Sectional Study
Source: Int J Nephrol. 2025 Dec 22;2025:5976864. doi: 10.1155/ijne/5976864 (PMC12752859; doi:10.1155/ijne/5976864)
Supplement: Supplementary file 1 — Supporting Information Additional supporting information can be found online in the Supporting Information section. [file IJNE-2025-5976864-s001.docx]

**Supplementary Table 1. Adjusted regression models for generic scales of SF-36 questionnaire**

|  |  | **Physical Functioning (PF)** | **Role-Physical (RP)** | **Bodily Pain(BP)** | **General Health(GH)** | **Vitality** | **Role-Emotional (RE)** | **Social Functioning (SF)** | **Fatigue/energy** |
| --- | --- | --- | --- | --- | --- | --- | --- | --- | --- |
| Group^ | Transplantation | 45.8  (-48.9-140.5) | 2.74  (-66.9-72.4) | **26.6**  **(7.1-46.1)*** | **41.6**  **(7-76.1)*** | **43.4**  **(5.2-81.6)*** | -11.4  (-64.5-41.4) | 5.3  (-14.1-24.8) | 28.7  (-3.7-61.2) |
|  | PD | -28.3  (-128.4-71.8) | 66.7  (-6.2-139.6) | -5.6  (-26.1-14.8) | 28.9  (-7.5-65.3) | 3.4 (-36.4-43.2) | **-57.8**  **(-113.1 to -2.7)*** | 7.35  (-12.8-27.6) | 6.4  (-27.1-40.3) |
| Age | | -5.3  (-7.6 to -3)* | -2.88  (-4.6 to -1.1)* | -0.85  (-1.3 to -0.4)* | -1.15  (-2.1 to – 0.3)* | -0.43 (-1.34-0.5) | -0.9  (-2.304-0.33) | -0.73  (-1.2 to -.23)* | -1.1  (-1.9 to -0.4)* |
| BMI | | -8.5  (-18.1-1.1) | -8.9  (-15.9 to -1.8)* | -0.3  (-2.3-1.6) | -2.24  (-5.8-1.3) | -1.7  (-5.6-2.1) | -1.7  (-7.1-3.7) | -2.1  (-3.9 to -0.34)* | -2.8  (-6.1-0.3) |
| Income | | -0.73  (-0.14-0.01) | -0.5  (-0.1-0.001) | -0.1  (-0.01-0.01) | -0.04  (-0.7 to -0.01)* | -0.01  (-0.04-0.01) | 0.007  (-0.3-0.04) | -0.0  (-0.2-0.004) | -0.02  (-0.5 to -0.003)* |

*p<0.05; PD: Peritoneal Dialysis; BMI: body mass index

^ HD is sat as a reference group

**Supplementary Table 2. Adjusted regression models for specific scales of KDQOL-SF**

|  | | Symptom/problems | Effects of kidney disease on daily life | Burden of kidney disease | Work status | Cognitive function | Quality of social interaction | Sexual function | Sleep | Social support | Dialysis staff encouragement | Patient satisfaction |
| --- | --- | --- | --- | --- | --- | --- | --- | --- | --- | --- | --- | --- |
| **Group^** | **Transplantation** | 34.2(-37.2-105.1) | **155.4(87.1-223.7)*** | 36.28(-2.3-74.9) | -5.2(-27.6-17.1) | 2.01(-18.1-22.1) | 10.5(-12.9-34.1) | **36.1(2.1-69.8)*** | 3.6(-26.5-33.8) | 5.9(-14.2-26.1) | 15.1(-0.1-30.7) | -1.2(-10.9-8.4) |
|  | **PD** | -13.5(-87.2-60.2) | 87.2(19.1-155.3) | -12.7(-53.1-27.6) | -4.4(-27.6-18.8) | -18.9(-39.8-1.9) | -19.3(-43.9-5.2) | 1.3(-30.4-33.1) | -29.1(-60.5-2.4) | -16.3(-37.7-5.1) | 12.03(-3.4-27.4) | -15.2(-25.4 to -5.1)* |
| **Age** | | -3.3)-5.1 to -1.6)* | -0.11(-1.7-1.5) | -1.1(-2.1 to -0.22)* | -0.18(-0.73-0.37) | -0.33(-0.8-0.1) | -0.3(-0/9-0.2) | -0.5(-1.4-0.27) | -1.3(-2.1 to -0.6)* | -0.43(-0.9-0.07) | 0.19(-0.17-0.56) | 0.12(-0.11-0.37) |
| **Income** | | 0.003(-0.05-0.05) | -0.02(-0.07 to 0.02) | -0.002(-0.03-0.02) | 0.006(-0.01-0.02) | -0.2(-0.3 to -0.004)* | -0.02(-0.03 to -0.001)* | -0.01(-0.04-0.008) | -0.01(-0.03-0.01) | 0.01(-0.005-0.02) | 0.01(0.0001-0.02) | -0.001)-0.009-0.006) |
| **BMI** | | -0.27(-7.3-6.7) | -7.3(-13.7 to -0.8) | -0.9(-4.8-3.1) | -1.1(-3.4-1.1) | -0.2(-2.3-1.7) | -0.7(-3.1-1.7) | 0.5(-3.01-4.1) | 0.3(-2.7 to -0.6) | 1.1(-0.9-3.1) | 0.13(-1.3-1.6) | -0.5(-1.4-0.5) |

*p<0.05; PD: Peritoneal Dialysis; BMI: body mass index

^ HD is sat as a reference group
